# The Combined Administration of Eicosapentaenoic Acid (EPA) and Gamma-Linolenic Acid (GLA) in Experimentally Induced Colitis: An Experimental Study in Rats

**DOI:** 10.3390/jcm13226661

**Published:** 2024-11-06

**Authors:** Orestis Ioannidis, Angeliki Cheva, Ioannis Varnalidis, Ioannis Koutelidakis, Vasileios Papaziogas, Panagiotis Christidis, Elissavet Anestiadou, Konstantinos Aggelopoulos, Ioannis Mantzoros, Manousos George Pramateftakis, Efstathios Kotidis, Barbara Driagka, Stamatios Aggelopoulos, Evangelos J. Giamarellos-Bourboulis

**Affiliations:** 1 4th Department of Surgery, General Hospital “George Papanikolaou”, Aristotle University of Thessaloniki, 57010 Exochi, Greece; varnalidis@me.com (I.V.); panagiotischristidis13@gmail.com (P.C.); elissavetxatz@gmail.com (E.A.); kostaggelo@hotmail.com (K.A.); imanvol@gmail.com (I.M.); mpramateftakis@hotmail.com (M.G.P.); skotidis@gmail.com (E.K.); valiadrg@gmail.com (B.D.); saggelopoulos@auth.gr (S.A.); 2 Pathology Department, Faculty of Medicine, Aristotle University of Thessaloniki, 54124 Thessaloniki, Greece; antacheva@yahoo.gr; 3 2nd Department of Surgery, G.Gennimatas General Hospital, Aristotle University of Thessaloniki, 54635 Thessaloniki, Greece; iokoutel@auth.gr (I.K.); papaziog@auth.gr (V.P.); 4 4th Department of Internal Medicine, National and Kapodistrian University of Athens Medical School, “Attikon” Hospital, 12462 Athens, Greece; egiamarel@med.uoa.gr

**Keywords:** fatty acids, eicosapentaenoic acid (EPA), gamma-linolenic acid (GLA), ulcerative colitis

## Abstract

**Background/Objectives:** Ulcerative colitis (UC) is a chronic inflammatory bowel disease with limited effective treatments, prompting the need for investigation of novel therapeutic approaches. Eicosapentaenoic acid (EPA) and gamma-linolenic acid (GLA) have demonstrated potential anti-inflammatory properties, but their combined effects on UC have not been thoroughly investigated. This study aimed to evaluate the effect of the combined administration of EPA and GLA on clinical and histopathologic features of experimental UC models. **Methods:** Thirty-six male Wistar rats were randomized in three groups (DSS group, Ensure Plus group, and Oxepa group), with twelve rats in each group. Experimental colitis was induced by administrating dextran sulfate sodium (DSS) 8%. The DSS group received tap water, the Ensure Plus group was given a high caloric diet, and the Oxepa group received a special diet containing high levels of EPA and GLA. Disease activity index (DAI) and microscopic activity index (MAI) were measured. Inflammatory markers were calculated both in blood and large intestine, liver, spleen, and lung tissue samples. Neutrophil and macrophage populations were assessed with immunohistochemistry. **Results:** No significant differences in the DAI index were found between the groups, but the MAI revealed statistically significant differences (*p* < 0.001). While no significant differences were observed in tumor necrosis factor-alpha (TNF-α) levels, interleukin-17 (IL-17) levels in the large intestine showed statistically significant differences (*p* = 0.05), with the Ensure Plus and Oxepa groups displaying lower levels compared to the DSS group (*p* = 0.021 and *p* = 0.043, respectively). Significant differences in neutrophil infiltration were found in both the large intestine (*p* < 0.001) and lungs (*p* = 0.002), with the Oxepa group showing fewer cells. Similarly, significant differences in macrophage infiltration were observed in the large intestine (*p* = 0.038) and spleen (*p* < 0.001), with the Oxepa group having lower macrophage counts. **Conclusions:** In conclusion, the combination of EPA and GLA demonstrates local anti-inflammatory effects and improves the histopathological outcomes in UC.

## 1. Introduction

Ulcerative colitis (UC) is a chronic, diffuse inflammatory disease that affects the rectal and colon mucosa [1,2,3,4]. Patients with UC have a cytokine imbalance that leads to the development of inflammation, as several cytokines that promote the inflammatory response, including tumor necrosis factor-a (TNF-α) and interleukin-17 (IL-17), are elevated [5,6]. The increased incidence of idiopathic inflammatory diseases is associated with increased dietary intake of Ω6 polyunsaturated fatty acids (PUFAs). Therefore, increasing the ratio of Ω3/Ω6 PUFAs or adding Ω6 with anti-inflammatory properties may have therapeutic effects. Ω3 PUFAs are incorporated into the intestinal mucosa of patients with inflammatory bowel disease (IBD) receiving supplemental fish oil and result in anti-inflammatory activity with reduced leukotriene B (LTB4) production by neutrophils and the intestinal mucosa, reduced production of prostaglandin E_2_ (PGE2) and thromboxane B2 (TXB2) by the intestinal mucosa and reduced PGE-2 and interferon gamma (IFN-γ) production by monocytes [7,8,9,10,11]. Several new therapeutic approaches, including specific dietary solutions such as anti-inflammatory Ω3 and Ω6 PUFAs, aim to modify the immune response of the gut and restore the function of the intestinal mucosal barrier and flora [12,13,14].

The addition of dietary supplements containing Ω3 PUFAs has been shown to alleviate symptoms and improve the course of inflammatory diseases [15,16,17]. Eicosapentaenoic acid (EPA), an Ω3 PUFA, exerts anti-inflammatory activity by reducing the production of pro-inflammatory cytokines (TNF-α, IL-1β, IL-6) and oxidative stress, promoting the production of anti-inflammatory lipid agents such as resolvins and protectins, reducing oxidative stress and modifying of the production of eicosanoids [18,19,20,21,22,23]. The latter promote the inflammatory response by competing with arachidonic acid (AA) to inhibit the formation of Ω6 eicosanoids and chemotactic products that promote the inflammatory response and suppress cell-mediated immune responses [15,16,17]. In addition, EPA suppresses leukocyte activity, cell proliferation, pro-inflammatory cytokine synthesis, natural killer cell activity, antigen synthesis, and surface macrophage expression, and acts as a free radical scavenger, increasing the production of anti-inflammatory cytokines such as thromboxane A2, prostaglandin E3, and leukotriene B5 [15,16,17]. Gamma-linolenic acid (GLA) is a Ω6 PUFA that has anti-inflammatory properties and improves the clinical course of inflammatory diseases. Experimental and clinical studies have shown that GLA plays an important role in the development of the inflammatory response by competing with the activity of arachidonic acid (AA), thereby inhibiting the formation of its eicosanoids, which promote the inflammatory response, and increasing the production of prostaglandin E1, which has potent anti-inflammatory activity [24].

Dietary factors, including dietary lipids, particularly PUFAs, play an important role in modifying the inflammatory response and may influence not only the development but also the clinical course of IBD [25]. PUFAs play a role in regulating the inflammatory response through the production pro-inflammatory mediators called eicosanoids [26]. Plasma concentrations of PUFAs are related to levels of mediators that promote the inflammatory response. Ω6 PUFAs, with the exception of GLA, are generally thought to promote the inflammatory response, whereas Ω3, including EPA, are thought to have anti-inflammatory activity [22,27]. Total EPA levels are associated with lower levels of inflammatory markers (IL-6, TNFα, and C-reactive protein) and higher levels of anti-inflammatory markers (IL-10 and TGFβ), and therefore EPA is beneficial in patients with diseases where there is active inflammation [28]. The uptake of anti-inflammatory GLA and EPA is thought to be an important modifier of UC activity, and patients have been found to have significantly elevated levels of alpha-linolenic acid (ALA) and AA and significantly reduced levels of EPA both in blood serum and locally in the intestinal mucosa [29,30,31]. EPA supplementation is known to exert anti-inflammatory effects and reduce inflammatory markers in plasma and cells of patients with acute and chronic diseases. The beneficial properties of both PLA and GLA, particularly in limiting the inflammatory response, have been documented in a number of clinical and experimental studies and could therefore be used in the treatment of UC [32].

The observation that EPA has anti-inflammatory effects led to the idea that their absolute or relative deficiency may contribute causally to inflammatory conditions and that their administration to patients with inflammatory diseases may be of clinical benefit [33]. The anti-inflammatory effect of the combination of EPA and GLA has been successfully tested in the treatment of patients with acute respiratory distress syndrome, where in particular the combination of EPA with GLA reduced the number of neutrophils and amount of inflammation in the lungs [34]. In addition, administration of a combination of EPA and GLA to patients with asthma reduced the biosynthesis of leukotrienes [35].

Recent research has highlighted the interaction between PUFAs and the melanocortin system, particularly in the context of IBD [36]. The melanocortin system, which includes receptors, such as melanocortin 3 receptor (MC3R) and melanocortin 5 receptor (MC5R), plays a key role in modulating inflammation [37]. Studies have shown that these receptors are expressed at higher levels in inflamed gut segments, particularly in the colon, during IBD [38]. The anti-inflammatory properties of PUFAs may influence the activity of the melanocortin receptors, suggesting a potential therapeutic link between dietary PUFAs and the regulation of inflammation via the melanocortin pathway [38]. This emerging area of research warrants further exploration, as it may provide new insights into the management of IBD. In addition, it is important to note that PUFAs, particularly omega-3 fatty acids, have been shown to downregulate pro-inflammatory cytokines, which are also influenced by the melanocortin pathway. The anti-inflammatory actions of the melanocortin receptors, such as MC3-R and MC5-R, extend beyond the gut, playing a broader role in immune modulation [38]. In particular, MC3-R activation has been associated with the suppression of macrophage activity and reductions in TNF-α and IL-6, key drivers of inflammation in IBD [39]. The interaction between dietary PUFAs and melanocortin receptors may thus offer a dual mechanism of action, combining the systemic anti-inflammatory effects of PUFAs with localized control of intestinal inflammation through the melanocortin system. This convergence of pathways underscores the therapeutic potential of targeting both dietary and receptor-mediated approaches in managing the chronic inflammation seen in IBD.

IL-17 is a cytokine that promotes the inflammatory response and is mainly produced by specialized Th17 lymphocytes, and the important stimuli for its production are TNF-α and endotoxin [40,41,42,43,44]. However, it is also produced by many innate immunity cells, including neutrophils and macrophages [40,45]. In particular, IL-17 induces the production of secondary cytokines that promote the inflammatory response such as TNF-α, chemokines, growth factors, and adhesion molecules such as ICAM-1 from mesenchymal stromal cells, ultimately leading to local recruitment and activation of neutrophils locally and to an enhancement in tissue inflammation [40,46]. IL-17 plays a key role in the development of UC [47,48] and in experimentally induced colitis following DSS administration [49,50,51,52]. In IBD, lymphocyte infiltration in the mucosa and IL-17 expression are observed in the inflammatory mucosa of patients with active disease, in contrast to the absence of IL-17 in normal mucosal samples [43,47]. There is also increased synthesis of IL-17 mRNA in the intestinal mucosa of these patients, and elevated blood serum levels are found in patients with active IBD [53,54]. Both serum and mucosal IL-17 levels appear to be related to disease activity. The presence of IL-17 in the intestinal mucosa leads to the production of inflammatory cytokines (IL-6, IL-8, MCP-1) and matrix metalloproteinases [46]. In the inflammatory bowel environment of IBD, the major role of IL-17 may be to promote additional inflammatory cascades by stimulating and producing chemokines that recruit and activate granulocytes [5].

The primary objective of this study is to evaluate the efficacy of combined administration of EPA and GLA on the clinical and histopathological features of experimentally induced colitis in rats. This study aims to provide new insights into the local and systemic anti-inflammatory effects of EPA and GLA, which have not been extensively studied in combination. We expect that co-administration of these compounds will demonstrate a reduction in inflammatory markers such as TNF-α and IL-17 and improve histopathological outcomes. This research extends previous studies by focusing on both local and systemic inflammatory responses and providing a detailed comparison with high-calorie dietary interventions.

## 2. Materials and Methods

### 2.1. Experimental Animals

The study was a randomized, double-blind, prospective, experimental study in rats. The experiment and data reporting were conducted and presented in accordance with the ARRIVE (Animal Research: Reporting of in vivo Experiments) guidelines [5]. Thirty-six (36) white male Wistar rats weighing 350–500 g and aged 20–24 weeks were used as animal models. All animal models were housed in five-position metal cages under controlled environmental conditions with a temperature between 20 and 25 °C, relative humidity between 55 and 65%, and a 12 h light/dark cycle. The animal models had access to tap water and food ad libitum. The protocol was approved by the Central Macedonian Veterinary Directorate (Protocol No. 153775/931-08.06.2011). All methods were performed according to national legislation (Presidential Decree 160/91), which is harmonized with the requirements of the European Directives 86/609/EEC and 2007/526/EC (L197).

### 2.2. Groups of Animal Models

The animal models were randomly divided into 3 groups by means of block randomization [55]:

− DSS group, treated with 8% DSS and 3 mL of tap water every 12 h;
− Ensure Plus group, treated with 8% DSS and 3 mL of high calorie specific dietary supplement (Ensure Plus) every 12 h;
− Oxepa group, treated with DSS 8% and 3 mL of specific dietary solution containing a combination of Ω3 PUFA (EPA) and Ω6 PUFA (GLA) every 12 h.

Colitis was induced with 8% dextran sulphate sodium (DSS) dissolved in tap water according to an established protocol. At time 0, both the administration of the 8% DSS solution and the treatment began. At the end of day 8, at time 184, sedation and blood sampling were performed, followed by euthanasia. To ensure reproducibility, colitis was confirmed by clinical assessment (weight loss, stool consistency, and rectal bleeding) and histological examination. Inclusion criteria included male Wistar rats between 350 and 500 g, while exclusion criteria included animals showing excessive distress or lack of colitis based on histopathological evaluation.

### 2.3. Dextran Sulfate Sodium—Induction of Colitis

Dextran sulfate sodium (DSS) (TdB Consultancy, Upsala, Sweden—CAS number 9042-14-2) with a molecular weight of 42,844 (35,000–50,000) was used for the induction of colitis. DSS 8% aqueous solution (*w*/*v*) was prepared daily by dissolving DSS in tap water using a magnetic stirrer (Heidolph Instruments GmbH & Co. KG, Schwabach, Germany). Experimental colitis was induced in all animals by feeding them instead of tap water the 8% DSS solution.

### 2.4. EPA and GLA

The dietary formulation used as the source of EPA and GLA was Oxepa (Abbott Laboratories B.V., Zwolle, The Netherlands). Oxepa is a complete, balanced, low-carbohydrate, high-fat liquid diet containing high levels of EPA (4.6 g per liter), GLA (4 g per liter) and increased levels of antioxidants. Its caloric efficiency is 1.5 calories per milliliter (Kcal/mL) (Supplementary Materials—Table S1).

### 2.5. High-Calorie Diet

The nutrient formulation used as a high calorie diet was Ensure Plus (Abbott Laboratories SA, Athens, Greece). Its caloric efficiency is the same as Oxepa (1.5 calories/milliliter). Ensure Plus is a high-calorie, complete, balanced, protein-rich liquid dietary supplement. It contains a high concentration of EPA (alpha-linolenic acid—ALA 2.75 g/L), probiotic fibre (short-chain fructooligosaccharides—12 g/L) and increased levels of antioxidants (Supplementary Materials—Table S1).

### 2.6. Dietary Administration

Nutrition was administered by placing an orogastric catheter under sedation, where the animal was placed in a special glass bell for 2 min and exposed to an ether-soaked gauze.

### 2.7. Clinical Monitoring

A disease activity index (DAI) was determined by assessing the degree of daily weight loss, stool consistency (normal stools, loose stools, and diarrhea), and stool hemoccult positivity or gross hemorrhage, according to the method described by Murthy et al. and shown in Table 1 [56,57].

### 2.8. Anesthesia

The anesthesia procedure was performed after the experimental animal was intoxicated with a volatile anesthetic (ether). The procedure was carried out by placing the rat in a special glass bell containing a gauze impregnated with ether for 2 min. The animals were then anaesthetized by intraperitoneal administration of chloral hydrate at 400 mg/kg body weight.

### 2.9. Blood Sampling

Sacrifice took place at the end of day 8 and was performed under aseptic conditions. Once the animal’s abdomen had been shaved, washed, and cleaned with 10% iodine povidone, a midline laparotomy was performed with a 4 cm incision from the level of the xiphoid process. After dissection of the skin and rectus abdominis muscles, the portal vein and inferior vena cava were identified at the entrance to the peritoneal cavity. Blood samples were taken from the portal vein and inferior vena cava in all animals. Blood was collected from the portal vein using a sterile syringe with a 25 G diameter needle and from the inferior vena cava using a 23 G diameter needle. Samples were collected in 5 mL (mL) endotoxin-free tubes (BD Vacutainer, SST II Advance, Beckton, Dickinson and Company, Plymouth, UK) where they were centrifuged, and the supernatant was stored in sterile 2 mL Eppendorf tubes (Nantong Hailun Bio-Medical Apparatus Manufacturing Co., Ltd., Haimen, Jiangsu, China) at −70 °C until measurements were made.

### 2.10. Tissue Sampling

After blood sampling, liver, spleen, lung, and colon samples were collected and placed in specially sterilized 2 milliliter (mL) tubes for cold storage (Eppendorf, Athens, Greece), then immediately cooled in liquid nitrogen and kept at −70 °C for cytokine determination. The liver, spleen, lung, and colon were removed and placed in formalin solution for further pathological and immunohistochemical examination. Specifically, the colon from the ileocecal valve to the anal canal was dissected and removed, and the peripheral colon was opened.

### 2.11. Euthanasia

At the end of the procedure, the animals underwent intracardiac administration of 10% potassium chloride (KCL).

### 2.12. Histopathological Examination

Tissues were fixed in 10% formalin solution, dehydrated, and embedded in paraffin cubes. Histological sections of 3 micrometers (µm) thickness were taken from each cube and stained with hematoxylin–eosin. From the colon, the last 3 cm proximal to the anus were taken. In the peripheral intestine, from the translocation of the squamous epithelium to the cylinder, the microscopic activity index (MAI) was measured after hematoxylin–eosin staining in 15 randomly selected fields per sample, as shown in Table 2 [58]. The MAI for each animal was the mean of these 15 measurements. Tissue pathology was performed on the remaining tissues.

### 2.13. Tissue Homogenization

Tissue homogenization was performed as follows: the tissue was first weighed in a special sterile 2 mL tube (mL) (1st measurement). The tissue was then removed and the tube alone was weighed, as some tissue and blood were likely to remain in the tube (2nd measurement). The weight of the tissue was then calculated by subtracting the first measurement from the second. It was then homogenized with 1 mL saline and centrifuged at 3500× *g* rpm for 10 min. Finally, the supernatant was collected and stored at −20 °C.

### 2.14. TNF-α Measurement

Serum TNF-α was determined by a method based on the cytotoxic effect of TNF-α on the L929 fibroblast line (ATCC) [59,60]. This method was chosen over the immunosorbent ELISA method because of its ability to determine the total bioactive molecules of TNF-α [61].

### 2.15. IL-17 Measurement

Serum IL-17 was measured by enzyme-linked immunosorbent assay (ELISA) according to the manufacturer’s instructions (eBioscience, San Diego, CA, USA) [62].

### 2.16. Antibodies

For immunohistochemical staining, the polyclonal antibody MPO (DAKO, Glostrup, Denmark) was used to detect polymorphonuclear cells and the monoclonal antibody CD68, clone KP1 (DAKO, Glostrup, Denmark) was used to detect macrophages. The dilution of the antibodies was 1/200 for MPO and 1/50 for CD68.

### 2.17. Immunohistochemistry

Serial 3μ-thick sections were taken from each cube. Prior to immunohistochemistry, the tissue sections were kept in an oven at 60 °C for 18 h. Sections were deparaffinized with dewax solution in an automated immunohistochemical staining machine. Immunohistochemical staining was performed on a LEICA BOND automated machine with a two-step, full detection, short-chain biotin-free immunohistochemistry system (Bond polymer refine detection). Prior to antibody application, the antigen was retrieved at high temperature (99 °C) using 0.01 M citrate buffer (Bond epitope retrieval ER1) and EDTA pH = 8 (Bond epitope retrieval ER2) for 30 min. Antibody incubation was performed for 30 min at room temperature. The staining protocol, as performed by the automated machine, was initiated by incubation in hydrogen peroxide for 5 min, followed by washing 3 times for 1 min and incubation with primary antibody for 30 min at room temperature. This was followed by washing 3 times for 1 min and incubation with the secondary antibody for 20 min, followed by washing again 3 times for 1 min. The polymer was then incubated for 20 min and washed 3 times for 1 min. This was followed by incubation with a diaminobenzidine solution (3,3-diaminobeznidine—DAB), washing 3 times for 1 min, and staining with hematoxylin for 5 min. Finally, after dehydration in ascending order of alcohol (50th, 70th, 96th, and 100th × 5 min) and clarification on xylene twice for 10 min, a balm cover and caps were applied.

### 2.18. Morphometry

In the colon, inflammatory cells labelled with MPO and CD68 were counted in the experimental animals of the different groups using a slightly modified method described previously [63]. Briefly, 5–10 digital images at ×200 magnification were taken from each immunohistochemically labelled tissue section of each animal using a Nikon eclipse 50i microscope and a Nikon DS-5M-L1 digital system (Nikon, Tokyo, Japan).

From these photographs, which contained the various fields in which the highest possible positive cell counts were detected, 3 were randomly selected per animal model. Images from each experimental group were counted as positive cells per square millimeter (mm^2^) using the ImageJ (Version 1.54j 12 June 2024) image processing and analysis program (NIH, Bethesda, MD, USA) [64]. In the remaining tissues (liver, lung, and spleen), a semi-quantitative morphometric method was used to count positive cells labelled with MPO and CD68 in the experimental animals of the different groups. Specifically, in each animal model, the presence of positive cells in 10 representative sections was calibrated on a five-point scale based on the following classification: 0 (-): no positive cells; 1 (+): minimal positive cells; 2 (++): few positive cells; 3 (+++): multiple scattered positive cells; 4 (++++): confluent group of positive cells.

### 2.19. Statistical Analysis

Statistical analysis was performed using SPSS (Statistical Package for Social Sciences, version 19.0). We presented quantitative normally distributed variables as mean ± SD, while quantitative non-normally distributed variables are presented as median (range). Frequencies and percentages were used for nominal variables. ANOVA analysis with Bonferroni adjustment was used to investigate which variables with normally distributed data differed significantly between groups, while Kruskal–Wallis analysis was used to investigate which non-parametric variables differed significantly between groups. A *p*-value less than 0.05 was considered statistically significant.

## 3. Results

### 3.1. Disease Activity Index (DAI)

**Figure 1:**
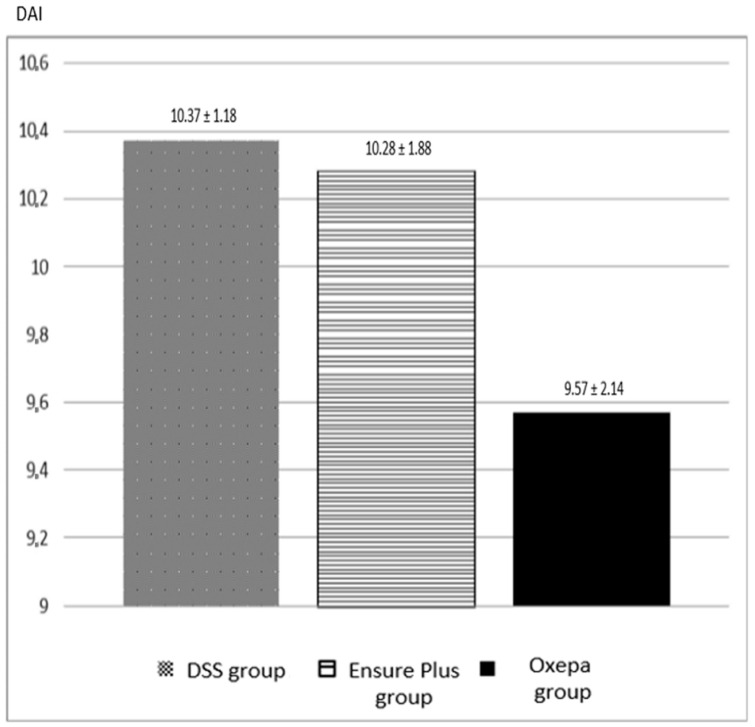
The disease activity index (DAI) of the different groups at the end of the experimental procedure. There was no statistically significant difference among groups.

During the experimental study, there was a gradual increase in the severity of the disease and consequently in the DAI in all groups (Figure 1). The peak was observed on day 8. From day 1 to day 2, a large proportion of the animal models exhibited loose stools, and by day 4 to day 5, diarrhea occurred in the majority of animals. Progressive weight loss was observed in all animals, starting on day 1. At the end of the study, all animals had diarrhea with hemorrhagic stools. The DAI was lower in the Oxepa group than in the Ensure Plus group and in the DSS group, which had the highest value. However, these differences were not statistically significant (*p* = 0.538) (Supplementary Materials—Table S2).

### 3.2. Microscopic Activity Index (MAI)

**Figure 2:**
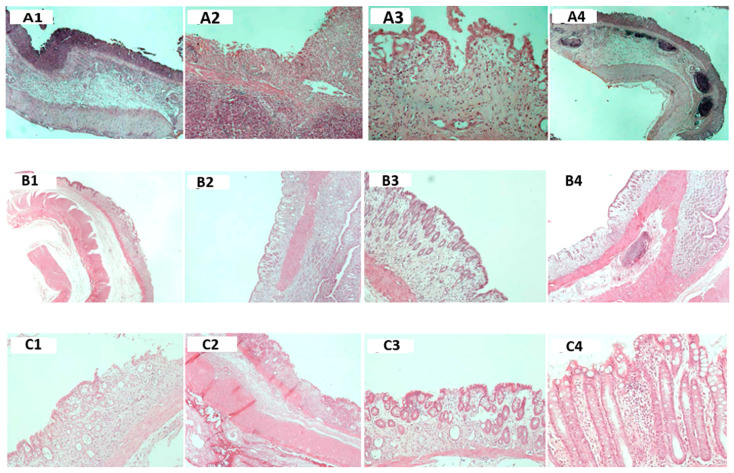
Microscopic findings of large intestine. (**A1**–**A4**) Microscopic findings of large intestine in DSS group. (**Α1**–**A3**) Mucosal fibrosis, inflammatory cell infiltrates in the presence of abundant neutrophilic and eosinophilic polymorphonuclear leukocytes, extensive loss of calycal cells, extensive loss of crypts, severe swelling, reactive and regenerative type of glandular epithelial lesions, extensive infiltration of the submucosal tissue by inflammatory cells with presence of multiple or large lymph nodes and vascular destruction [(**A1**) ×20, (**Α2**) ×40, (**Α3**) ×200]. (**A4**) Presence of multiple lymph nodes in the submucosa (×20). (**B1**–**B4**) Microscopic findings of large intestine in Ensure plus group. (**B1**–**B3**) Moderate loss of crypts with disarrangement of architecture and reduction of calyceal cells, extensive inflammatory cell infiltrates of the basement membrane, thickening of mucosal tissue due to edema, and limited ulceration with presence individual lymph nodes [(**B1**) ×20, (**B2**) ×40, (**B3**) ×100]. (**B4**): Presence of small ulcer and single lymph node in the submucosa (×40). (**C1**–**C4**) Microscopic small bowel findings in Oxepa group. (**C1**–**C3**) Limited loss of crypts and good maintenance of mucosal thickness and number of calyceal cells, limited inflammatory infiltration or absence there of submucosa and the mucosa mainly between the crypts and not inside it epithelium, and ulceration in the presence of fewer and smaller ulcers, with evidence of regeneration, absence of edema in the mucosa and submucosa, and absence of occasional presence of small lymphocytes. Even more rare ulcers with local regeneration; preservation of mucosal thickness; absence of edema and inflammation of the mucosa; and good preservation of crypts, goblet cells, and glands are observed, as well as mild inflammation between the crypts and not within the endothelium [(**C1**) ×40, (**C2**) ×100, (**C3**) ×100, (**C4**) ×200].

**Figure 3:**
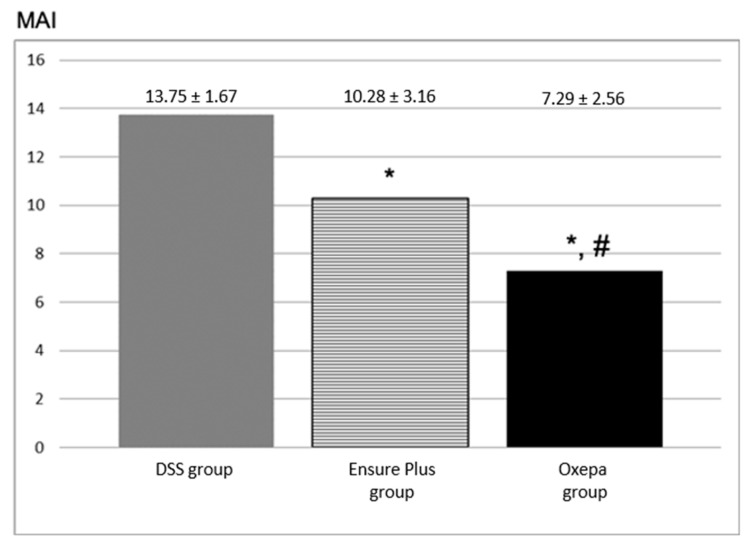
The microscopic activity index (MAI) of the different groups at the end of the experimental procedure. An asterisk (*) indicates a significant difference from DSS group, and hash (#) a significant difference from Ensure Plus group. Statistically significant differences were observed between the DSS and Ensure Plus groups (*p* = 0.011), DSS and Oxepa groups (*p* < 0.001), and Ensure Plus and Oxepa groups (*p* = 0.028).

Experimental colitis was observed histologically in all groups (Figure 2). The microscopic activity index was lower in the Oxepa group compared to the Ensure Plus group and the DSS group, which had the highest value (*p* < 0.001). The DSS group differed significantly from the Ensure Plus group (*p* = 0.011), the DSS group also differed from the Oxepa group (*p* < 0.001), and the Ensure Plus group differed from the Oxepa group (*p* = 0.028). These results are shown in Figure 3 (Supplementary Materials—Table S3).

### 3.3. Local Inflammatory Reaction

#### 3.3.1. TNF-α and IL-17 in Colon

TNF-α levels in the large intestine were lower in the Oxepa group compared to the DSS and Ensure Plus groups, which had the highest levels. However, these differences were not statistically significant. IL-17 levels were lower in the Ensure Plus group compared to the Oxepa and DSS groups, which had the highest levels. These differences were significant (*p* = 0.05), specifically between the DSS and Ensure Plus groups (*p* = 0.021) and between the DSS and Oxepa groups (*p* = 0.043), with no significant difference between the Ensure Plus and Oxepa groups (*p* = 0.729).

#### 3.3.2. Neutrophil Polymorphonuclear Leukocytes (MPO-Positive Cells) and Macrophages (CD68-Positive Cells) in the Colon

**Figure 4:**
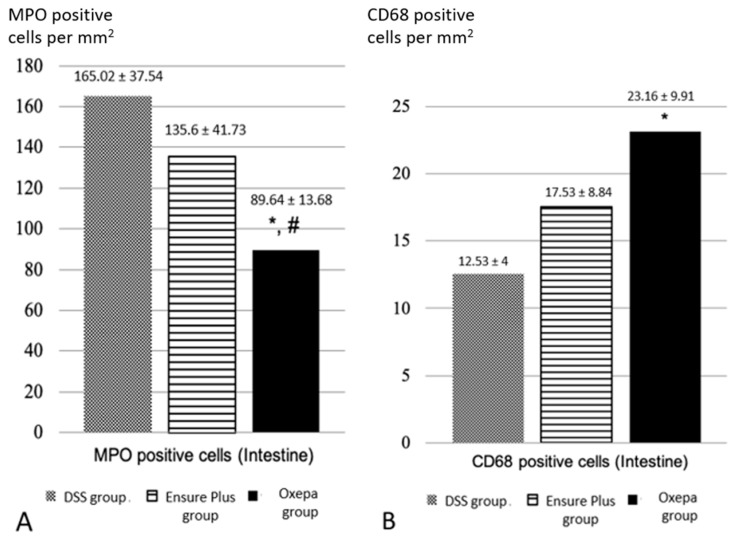
(**A**) The number of MPO positive cells (neutrophils) in the colon (per mm^2^) in the different groups at the end of the experimental procedure. An asterisk (*) indicates a significant difference from DSS group, and a hash (#) a significant difference from Ensure Plus group. (**B**) The number of CD68-positive cells (macrophages) in the colon (per mm^2^) in the different groups at the end of the experiment process. An asterisk (*) indicates a significant difference from DSS group.

The number of neutrophils in the large intestine, as indicated by MPO-positive cells, was reduced in the Oxepa group compared to both the Ensure Plus and DSS groups, which had the highest values (Figure 4A). These differences were significant (*p* < 0.001). While the difference between the DSS and Ensure Plus groups was not statistically significant (*p* = 0.077), the differences between the DSS and Oxepa groups (*p* < 0.001) and between the Ensure Plus and Oxepa groups (*p* = 0.007) were significant.

**Figure 5:**
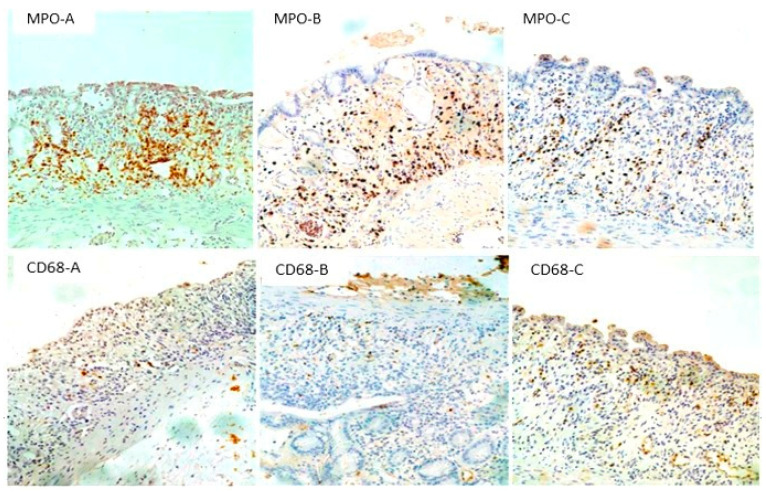
(**MPO A**–**C**) MPO immunohistochemical staining of colon tissue sections. Numerous positive cells (neutrophils) were observed in both DSS ((**MPO-A**) ×200) and Ensure Plus groups ((**MPO-B**) ×200), whereas in Oxepa group statistically significant fewer positive cells ((**MPO-C**) ×200) were observed. (**CD68 A**–**C**) Immunohistochemical staining of CD68 of colon tissue. A small number of positive cells (macrophages) are observed in the DSS group ((**CD68-A**) ×200), whereas in the Oxepa group, there are statistically significant more positive cells ((**CD68-C**) ×200). Ensure Plus group B showed an intermediate number of positive cells that did not differ significantly from the other 2 groups ((**CD68-B**) ×200).

The number of macrophages in the large intestine, as indicated by CD68-positive cells, was higher in the Oxepa group compared to the Ensure Plus and DSS groups, which had the lowest values (Figure 4B). The difference between the DSS and Oxepa groups was significant (*p* = 0.011), but not between the DSS and Ensure Plus groups (*p* = 0.185), nor between the Ensure Plus and Oxepa groups (*p* = 0.149). These results are shown in Figure 5 (Supplementary File Tables S4 and S5).

### 3.4. Systemic Inflammatory Reaction

#### 3.4.1. TNF-α and IL-17 in the Inferior Vena Cava and Portal Vein Serum

**Figure 6:**
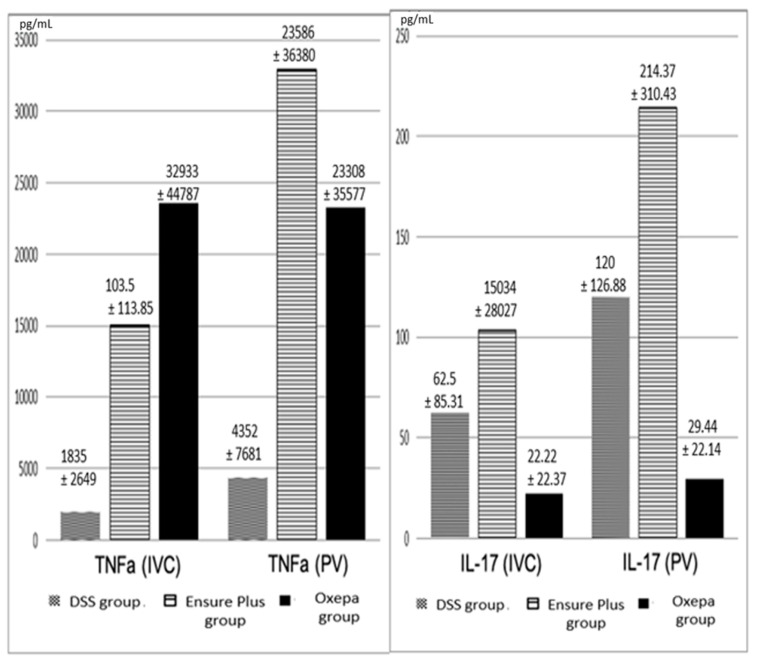
Values of TNF-α (pg/mL) and IL-17 (pg/mL) in the inferior vena cava (IVC) and portal vein (PV) in the different groups at the end of the experimental procedure. Despite the obvious differences, *p* is not statistically significant.

TNF-α levels in both the inferior vena cava and portal vein were lower in the DSS group compared to the Ensure Plus and Oxepa groups. However, these differences were not statistically significant. However, IL-17 levels were lower in the Oxepa group compared to the DSS and Ensure Plus groups. However, these differences were not statistically significant. These results are shown graphically in Figure 6 (Supplementary Materials—Tables S6 and S7).

#### 3.4.2. TNF-α and IL-17 in Liver, Spleen, and Lung Tissues

**Figure 7:**
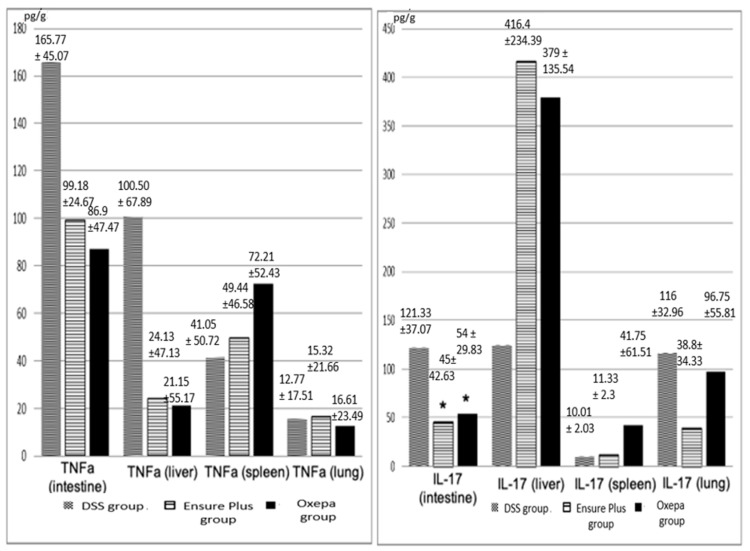
TNF-α (pg/g) values in tissues in the different groups at the end of the experimental procedure. Despite the obvious differences in some tissues, *p* is not statistically significant. IL-17 (pg/g) values in tissues in the different groups at the end of the experimental procedure. An asterisk (*) indicates a significant difference between groups.

TNF-α levels in both the inferior vena cava and the portal vein were lower in the DSS group compared to the Ensure Plus and Oxepa groups, but these differences were not statistically significant. Similarly, IL-17 levels were lower in the Oxepa group compared to the DSS and Ensure Plus groups, but again these differences were not statistically significant. These results are shown in Figure 7 (Supplementary Materials—Tables S8 and S9).

#### 3.4.3. Neutrophilic Polymorphonuclear Leukocytes (MPO-Positive Cells) and Macrophages (CD68-Positive Cells) in Liver, Spleen, and Lung Tissues

**Figure 8:**
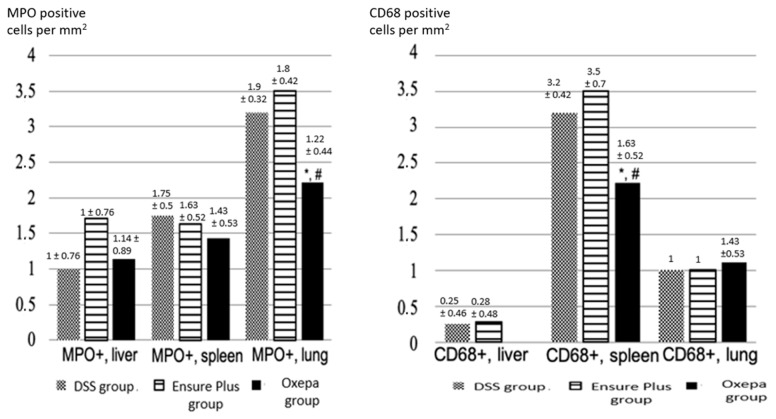
The presence of MPO-positive cells (neutrophils) in the liver, spleen, and lungs in the different groups at the end of the experimental procedure. An asterisk (*) indicates a significant difference from DSS group, and a hash (#) a significant difference from Ensure Plus group. Values of the degree of presence of CD68-positive cells (macrophages) in the liver, spleen, and lungs in the different groups at the end of the experimental process. An asterisk (*) indicates a significant difference from DSS group, and a hash (#) a significant difference from Ensure Plus group.

**Figure 9:**
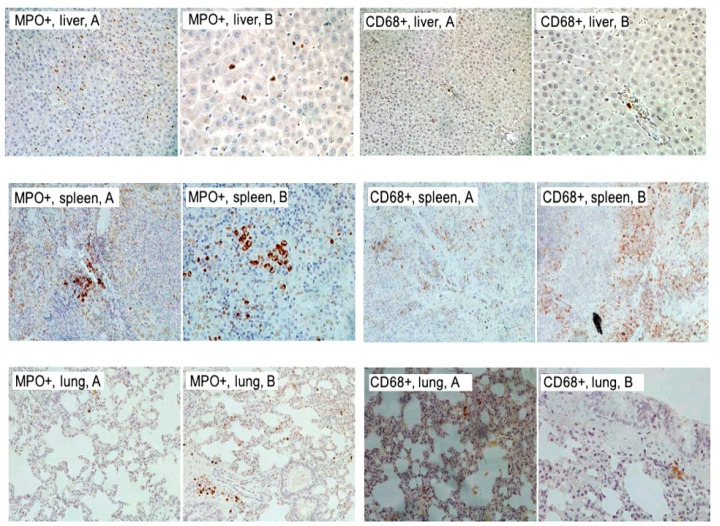
MPO and immunohistochemical staining of MPO and CD68 among various tissues. MPO immunohistochemical staining of liver tissue sections. A few neutrophils are depicted ((**A**) ×200, (**B**) ×400). Findings are similar across groups. Immunohistochemical staining of CD68 of liver tissue sections. The findings are similar in all groups, without the presence of positive cells. Rarely are macrophages illustrated ((**A**) ×200, (**B**) ×400). MPO immunohistochemical staining of spleen tissue sections. A few neutrophils in small groups are shown ((**A**) ×200, (**B**) ×400). Findings are similar across groups. CD68 immunohistochemical staining of spleen tissue sections. (**A**) Few macrophages (×200) are depicted, findings observed in group C. (**B**) A few to confluent macrophages are depicted (×200); findings observed in DSS and Ensure Plus groups. MPO immunohistochemical staining of lung tissue sections. (**A**) Rare neutrophils (×200), findings observed in Oxepa group. (**B**) Few neutrophils (×200), findings observed in DSS and Ensure Plus groups. CD68 immunohistochemical staining of lung tissue sections. Rare macrophages are depicted ((**A**) ×200, (**B**) ×400). Findings are similar across groups.

The presence of MPO-positive neutrophils in the liver was lower in the DSS group compared to the Ensure Plus and Oxepa groups, but this decrease was not significant (Figure 8 and Figure 9). In the spleen, MPO-positive cells were lower in the Oxepa group compared to the DSS and Ensure Plus groups, but the decrease was not significant. In the lungs, MPO-positive cells were also lower in the Oxepa group compared to the DSS and Ensure Plus groups. Rarely were any positive cells observed in the Oxepa group, while a few were present in the DSS and Ensure Plus groups, and this decrease was significant (*p* = 0.002). Specifically, the difference between the DSS and Ensure Plus groups was not significant (*p* = 0.576), but the decrease between the DSS and Oxepa groups (*p* = 0.001) and between the Ensure Plus and Oxepa groups (*p* = 0.004) was significant.

The presence of CD68-positive macrophages in the liver was lower in the Oxepa group compared to the DSS and Ensure Plus groups, but this decrease was not significant. In the spleen, CD68-positive cells were also lower in the Oxepa group compared to the DSS and Ensure Plus groups, which had the highest levels. Fewer positive cells were observed in the Oxepa group compared to the DSS and Ensure Plus groups, and this reduction was significant (*p* < 0.001). Specifically, the difference between the DSS and Ensure Plus groups was not significant (*p* = 0.227), but the decrease between the DSS and Oxepa groups (*p* = 0.001) and between the Ensure Plus and Oxepa groups (*p* < 0.001) was significant.

In the lungs, CD68-positive macrophages were higher in the Oxepa group compared to the DSS and Ensure Plus groups, but this increase was not significant (Supplementary Materials—Tables S10 and S11).

## 4. Discussion

In UC, the cytokine balance is disturbed, leading to the development of inflammation. The production of cytokines and chemokines that promote the inflammatory response is enhanced, while the expression of adhesion molecules and co-stimulatory molecules is increased [65]. In addition, the production of eicosanoids, leukotrienes, and free radicals is increased [66].

The present study was designed to investigate both the efficacy of co-administration of EPA and GLA in the treatment of experimental UC in rats and their effect on the local and systemic inflammatory response. The disease activity index (DAI) was used to study the clinical course, while the microscopic activity index (MAI) was determined after pathological examination of the rectum. The local inflammatory response was studied by measuring the levels of TNF-α and IL-17, as well as the neutrophil and macrophage population in the colon, while the systemic inflammatory response was studied by measuring the same parameters in the liver, spleen, and lungs of the rats, as well as measuring TNF-α and IL-17 in the lower vena cava and portal vein. Dextran sulphate sodium (DSS) was used to induce experimental ulcerative colitis. This protocol is a well-established model of inflammatory bowel disease that is clinically and histologically similar to UC [67,68,69]. The model predicted the development of UC by administration of 8% DSS in drinking water for 8 days and concurrent administration of 12 h of tap water, a special high-calorie diet (Ensure Plus), or a special dietary solution containing a combination of Ω3 PUFAs (EPA) and Ω6 PUFAs (GLA) (Oxepa) at a dose of 3 mL. Our results, especially in terms of UC inducement, allow for us to conclude that the model was successful, as it caused the clinical and microscopic manifestation of the disease in all experimental animals. Also, based on our clinical and histopathological results, we could say that the induced colitis had the character of acute severe colitis. It is the first model that has successfully administrated such a high dose of DSS with no mortality recorded, and one of the few models that test the therapeutic intervention during the development of UC and not before or after its development. Regarding the clinical activity of the disease, the results although showing improvement in the group receiving the combination of EPA and GLA, these are not statistically significant compared to the other two groups. In contrast, our results on the microscopic activity of the disease allow for us to conclude that administration of a combination of EPA and GLA was effective in treating UC both in comparison to tap water and to the specific dietary solution of high caloric yield, while this solution was also effective in relation to tap water.

Our results are partially consistent with those of Middleton et al. [70], who investigated the combined administration of EPA and GLA in patients with UC in the only randomized, double-blind, controlled clinical trial. The administration of GLA (1.62 g) in combination with EPA (0.27 g) and DHA (0.045 g) daily for 12 months did not result in a significant reduction in relapse rates compared to the control. There were also no significant differences in endoscopic findings and blood tests. The lack of positive results may be due to the low doses of PUFAs administered and the lack of pathological examination. In addition, the study by Greenfield et al. [25] compared EPA and GLA with placebo in a randomized, placebo-controlled trial. Patients with UC received one of the two treatments for 6 months in addition to their usual medical treatment, and stool composition, the presence of blood in the stool, and endoscopic and pathological findings were studied. There were no significant differences between the three groups, except for the fact that GLA improved stool composition. Furthermore, we could say that our results are in contrast to those of Bassaganya-Riera et al. [71], the only experimental study that investigated the effect of administering conjugated LLA, another anti-inflammatory GLA and EPA alone or in combination in pigs with DSS colitis. The animals were fed the PUFA-enriched diet for 49 days, while colitis was induced from day 42 by administration of 4% DSS for 7 days. The combined administration of EPA and GLA resulted in a statistically significant reduction in clinical disease activity compared to the control group only on day 7, whereas the single administration of EPA and GLA resulted in a reduction in clinical disease activity compared to the control group on days 6 and 7. In addition, on day 7, the GLA group had a statistically significantly lower activity than the EPA group and the combined administration group, which had approximately the same activity. In terms of histological findings, the GLA group had statistically significantly less mucosal thickening than all groups of essentially the same magnitude, the EPA and combined administration groups had statistically significantly increased regeneration compared to the other two groups, while all experimental groups had significantly reduced ulceration. Similarly, in the study by Cho et al. [72]—which, like our study, investigated therapeutic intervention during the development of colitis—co-administration of EPA, in the form of DHA, together with DSS 5% for 7 days in rats with acute experimental colitis resulted in a significant reduction in weight and length of the colon, but no significant improvement in stool composition and hematochezia. There was also a significant improvement in microscopic disease activity in the group receiving EPA.

Experimental induced colitis following DSS administration is a well-established model of IBD that is clinically and histologically similar to UC [68,69]. The cytokines TNF-α and IL-17 play an important role in its pathogenesis [49,50,51,52], while histologically induced colitis is characterized by mucosal infiltration with neutrophils and macrophages [73,74,75]. TNF-α is a key mediator of the acute inflammatory response, is chemotactic for neutrophils and macrophages, and plays an important role in the pathogenesis of UC [76,77,78,79,80,81,82,83,84] and in experimentally induced colitis following DSS administration [50,52,85]. In IBD, TNF-α is expressed at high levels in the basement membrane of the intestine. Our results from the determination of TNF-α levels in serum from the inferior vena cava and portal vein, as well as in the intestine, liver, spleen and lung, showed no statistically significant difference when comparing the groups. However, locally in the gut, there was a trend towards lower TNF-α levels in the Oxepa group compared to the other groups, but this did not reach statistical significance. It can therefore be concluded that the administration of a combination of EPA and GLA does not affect the local and systemic production of TNF-α, and the histological improvement observed is independent of this. Our results are in contrast to those of Bassaganya-Riera et al. [71], who observed significantly reduced expression of TNF-α mRNA in the colon in all groups compared to the control group, and also that the GLA group had significantly reduced expression compared to the other two experimental groups, which had essentially the same levels. These comparisons highlight the relevance of our findings and demonstrate the potential anti-inflammatory effects of EPA and GLA combinations. However, unlike previous studies, our work uniquely examines the co-administration of EPA and GLA, providing further evidence of their synergistic effects in reducing local inflammation.

Our results of measuring IL-17 levels in the colon showed a statistically significant decrease in colonic IL-17 levels in both the group receiving the EPA and GLA combination and the group receiving a high-calorie nutrient solution compared to the control group receiving tap water. IL-17 levels were not significantly different between these groups. Our results are partially consistent with the research of Cho et al. [72], where the EPA group showed a significant reduction in the expression of cytokine genes that promote inflammatory response. In contrast, IL-17 levels in inferior vena cava and portal vein serum, as well as liver, spleen, and lung, did not show a statistically significant difference when comparing the groups, although the administration of the combination of EPA and GLA caused a tendency to reduce IL-17 in both the portal and systemic circulation. Therefore, it can be concluded that the administration of the combination of EPA and GLA induced a local reduction of IL-17 in the colon, which led to a histological improvement in inflammation, but did not affect its systemic production. It is possible that the reduction in IL-17 is due to the presence of EPA, which influences the synthesis of inflammatory cytokines, a process that is partially regulated by the prostaglandin PGE2 and leukotrienes. EPA reduced the ex vivo production of TNF-α, IL-1β, and IL-6 by rodent macrophages [86,87]. In addition, administration of EPA to a cancer patient resulted in decreased production of cytokines that promote inflammatory responses (TNF-α, IL-1, and IL-6) [88]. GLA has also contributed to this, as dietary administration of GLA increases the amount of GLA produced by prolonged DGLA without a concomitant increase in AA. This is because the conversion of DGLA to AA is influenced by D5 desaturase, which has limited activity, so only a small proportion is converted to AA. Therefore, DGLA, but not AA, accumulates in the cell membrane of inflammatory cells [89]. This increase in DGLA relative to AA is able to reduce the production of AA eosinocytes by inflammatory cells, which promote the inflammatory response and therefore exert anti-inflammatory activity [15].

In UC, cellular filtration is widespread and extends to the deeper mucosal layers (transmucosal), and the presence of neutrophils, which indicate a change in the composition of the inflammatory infiltrate, is another important element. Neutrophils are inextricably linked to the development of inflammation in the colon and the degree of neutrophil infiltration is an indicator of the activity and intensity of UC [90,91,92,93]. Histologically, experimentally induced colitis after DSS administration is characterized by mucosal infiltration with neutrophils, which play an important role in causing damage to colonic tissue [73,94]. Our results of neutrophil determination—after specific myeloperoxidase (MPO) immunohistochemical staining—in the colon showed a statistically significant decrease in their number in the group receiving the combination of EPA and GLA compared to the control group receiving tap water, as well as in the group receiving the high calorie specific diet, while no statistically significant differences were observed between the two groups. In the study by Cho et al. [72], co-administration with DSS 5% for 7 days in mice with acute experimental colitis resulted in significantly reduced levels of myeloperoxidase in the colon. Similar results regarding the degree of neutrophil infiltration were also observed in the lungs, with a statistically significant decrease in the group receiving the combination of EPA and GLA compared to the control group receiving tap water and the group receiving the high-calorie specific diet, while no statistically significant differences were observed between the two groups. However, there were no statistically significant differences in the degree of neutrophil infiltration in the liver and spleen. It can therefore be concluded that the administration of a combination of EPA and GLA caused a reduction in local inflammatory neutrophil infiltration in the large intestine, which led to a histological improvement of inflammation, possibly due to a reduction in the inflammatory process and hence a reduction in its damaging effect in the mucosa. The combination of EPA and GLA also led to a reduction in the systemic inflammatory response by reducing inflammatory infiltration in the lungs, but not in the liver and spleen. The reduced neutrophil infiltration in group C seems to be related to the altered production of eicosanoids. The metabolism of AA by cyclooxygenases leads to the production of prostaglandins and thromboxanes. The increased consumption of EPA leads to increased proportions of these PUFAs in the cell membrane phospholipids of inflammatory cells, mainly at the expense of AA [16,17]. As there is less substrate available for the synthesis of eicosanoids from AA, increased consumption of EPA results in decreased production of PGE2, TXB2, LTB4, LTE4, and 5-HETE by inflammatory cells [10,74]. In addition, EPA competitively inhibits the oxygenation of AA by cyclooxygenase. The increased consumption of EPA results in increased production of PGE3, LTB5, LTE5, and 5-Hydroxyeicosapentaenoic acid [95]. The functional significance of this event is that mediators produced by EPA are often less potent than those produced by AA, or even anti-inflammatory. Specifically, the leukotriene LTB5 is 10 to 100 times less potent than LTB4 as a chemotactic factor for neutrophils [42].

Macrophages are essential cells of the body’s immune system and are found in the colon mucosa. They play a key role in controlling the inflammatory response in the mucosa. Macrophages are activated by the presence of antigens and can promote the inflammatory response by producing pro-inflammatory mediators such as TNF-α, which stimulates further proliferation and inflammatory infiltration by neutrophils and macrophages. In UC, macrophages are increased due to their pathological activation [42,96,97,98]. On the other hand, in mucosal inflammation, macrophages proliferate to combat microbial invasion and also play a key role in resolving the inflammatory response and promoting the healing process following mucosal injury. Although histologically induced colitis following DSS administration is characterized by long-term mucosal infiltration, this is found to be more elevated during the healing phase than during acute inflammation, confirming their key role in healing [73,94]. Thus, depending on the stimuli and local conditions, macrophages can promote or attenuate the inflammatory process. Our results from the identification of macrophages after specific immunohistochemical staining with CD68 in the colon showed a statistically significant increase in their number in the group receiving the combination of EPA and GLA compared with the control group receiving tap water. In addition, there was a statistically significant reduction in macrophages in the spleen in the group receiving the combination of EPA and GLA compared with the control group receiving tap water and the group receiving the high-calorie special diet. However, no statistically significant differences were observed in the degree of macrophage infiltration in the liver and lungs. It can therefore be concluded that the combination of EPA and GLA caused an increase in local macrophage infiltration into the colon, leading to a histological improvement of inflammation, possibly through increased phagocytosis of invading microorganisms and promotion of healing. In addition, we can conclude that the combination of EPA and GLA led to a limitation of the systemic inflammatory response, since it caused a significant reduction in macrophages in the spleen, but not in the liver and lungs, possibly by limiting bacterial translocation due to increased phagocytosis locally in the colonic mucosa by the increased macrophage population. The amelioration of the histopathological lesions associated with increased macrophage infiltration may be due to the fact that EPA, through the action of COX-2 cyclooxygenases and LOX lipoxygenases, a recently discovered group of mediators, produces E-series resolvins, which appear to exert potent anti-inflammatory activity on neutrophils, macrophages, dendritic cells, and T lymphocytes [4,99,100]. Resolvins are endogenous, locally mediated agents with potent anti-inflammatory and immunomodulatory properties. In particular, they reduce inflammatory infiltration, regulate the cytokine and chemokine axis, limit the production of reactive oxygen radicals, and reduce the extent of inflammation [16,101,102].

Similar to the EPA and GLA, numerous other natural compounds, including Hericium erinaceus, have emerged as potential modulators of TNF-α. The extract of Hericium erinaceus, or lion’s mane mushroom, has demonstrated an ability to reduce TNF-α and pro-inflammatory cytokines in experimental models, particularly in neural and colitis studies [103]. Comparatively, both Hericium erinaceus and the combination of EPA and GLA target key pro-inflammatory pathways [104,105]. In models of colitis, Hericium erinaceus has shown significant downregulation of TNF-α, alongside reductions in IL-6 and IL-1β, reflecting its role in mitigating inflammation similarly to EPA and GLA combinations [105,106]. Both substances exhibit multi-targeted anti-inflammatory effects, though Hericium erinaceus has been more prominently investigated in neurological and cognitive models, while EPA and GLA have had broader applications in metabolic and autoimmune diseases [107]. These findings highlight the growing recognition of Hericium erinaceus as part of a broader spectrum of natural compounds capable of modifying inflammatory responses, such as those involving TNF-α, akin to other polyunsaturated fatty acids like EPA and GLA [108,109]. Future studies could explore their combined effects in synergistic anti-inflammatory therapies, especially in chronic inflammatory diseases such as ulcerative colitis and neuroinflammatory disorders.

In addition to EPA, GLA, and Hericium erinaceus, several other natural substances have been shown to modulate TNF-α levels in experimental models. For instance, curcumin, the active compound in turmeric (Curcuma longa), is widely recognized for its anti-inflammatory properties [110]. Curcumin significantly reduces TNF-α levels in various disease models, including colitis, arthritis, and metabolic disorders [111,112]. It acts by inhibiting NF-κB, a critical transcription factor that regulates TNF-α production. Studies demonstrate that curcumin suppresses TNF-α in colitis models, leading to reduced intestinal inflammation and improved clinical outcomes [113]. Resveratrol, a polyphenol found in grapes and red wine, is another potent modulator of TNF-α [114]. In experimental models of inflammatory diseases, resveratrol reduces TNF-α and other pro-inflammatory cytokines by inhibiting key signaling pathways such as NF-κB and STAT3 [115,116]. In colitis models, resveratrol administration resulted in decreased TNF-α expression, reduction in inflammatory cell infiltration, and improved histological outcomes [117,118]. Resveratrol’s dual role as an antioxidant and anti-inflammatory agent makes it an attractive candidate for controlling inflammation in chronic diseases [119,120,121]. Green tea polyphenols, particularly epigallocatechin gallate (EGCG), have also shown promise in regulating TNF-α levels [119]. EGCG inhibits TNF-α production in inflammatory conditions, particularly in models of IBD and arthritis [120,122]. By modulating immune responses and inhibiting pro-inflammatory cytokines, EGCG has demonstrated significant potential in alleviating symptoms of colitis and other TNF-α-mediated diseases [123,124]. Another noteworthy substance is quercetin, a flavonoid found in various fruits and vegetables. Quercetin reduces TNF-α production in several inflammatory models, including colitis, cardiovascular disease, and rheumatoid arthritis. By inhibiting TNF-α, quercetin helps to attenuate the inflammatory cascade, resulting in decreased tissue damage and improved clinical parameters in animal models [125]. Collectively, these natural substances—curcumin, resveratrol, EGCG, and quercetin—share the ability to modulate TNF-α levels and exhibit anti-inflammatory effects in experimental models, similar to EPA, GLA, and Hericium erinaceus. These compounds act through diverse mechanisms, often converging on common pathways such as NF-κB, which is a central regulator of TNF-α production [126]. This reinforces the potential for combining multiple natural agents to synergistically target TNF-α and other pro-inflammatory mediators in chronic inflammatory diseases such as colitis.

In conclusion, our results show that the combination of EPA and GLA improves the histological picture of experimental ulcerative colitis, both in relation to the control group receiving tap water and to the group receiving the special diet containing also EPA (α-linolenic acid—ALA). This effect of PUFAs is related and to some extent can be attributed to local production of IL-17, which is statistically significantly reduced compared to the control group, but not affected by local TNF-α production. In addition, the improvement in the histological image following administration of the combination of EPA and GLA is related and to some extent can be attributed to the local decrease in neutrophil count and to the simultaneous increase in macrophage number in the large intestine. The decrease in the number of neutrophils probably caused a reduction in the inflammatory process and hence their harmful effect on the mucosa. The increase in the number of macrophages probably reduced the inflammation and promoted the healing process. In addition, it seems that the combination of EPA and GLA has little effect on systemic inflammatory response as TNF-α and IL-17 values may not differ significantly in liver, spleen, lung, systemic, and portal circulation, but infiltration from neutrophils is significantly reduced in the lungs but not in the liver and spleen, and macrophage infiltration is significantly reduced in the spleen but not in the liver and lungs, in both cases between the group receiving the combination of EPA and GLA compared with the other two experimental groups. Furthermore, the combination of the increased number of macrophages locally in the large intestine with their reduced number in the spleen after administration of the combination EPA and GLA is probably due to increased phagocytosis of invading mucosal microorganisms and to a reduction in bacterial translocation that led to an increase of macrophages in the spleen in the other two experimental groups. From the present data, we can deduce that the combination of EPA and GLA exerts significant local anti-inflammatory activity, improves the histological picture of ulcerative colitis, and, at the same time, it demonstrates mild systemic anti-inflammatory action. Considering the fact that it does not cause any particular side effects, it could act as a significant adjuvant drug in the battle against IBD and especially the UC.

## Figures and Tables

**Table 1:** Disease activity index (DAI) scoring system.

Score	Weight Loss (%)	Stool Composition	Rectal Hemorrhage
0	None	Normal	No evidence
1	1–5	-	-
2	6–10	Soft (semi-formed stool)	-
3	11–15	-	-
4	>15	Diarrhea	Macroscopic hemorrhage

**Table 2:** Microscopic activity index (MAI) scoring system.

Score	Epithelium	Infiltration	Ulceration	Lymph Node Activity
0	Normal	None	None	None
1	Goblet cells loss	Focal infiltration around the basis of the crypts	One ulcer	One lymph node
2	Extensive loss of goblet cells, epithelium thinning	Extensive infiltration of the stratum basale	Two ulcers	Two lymph nodes
3	Loss of crypts, architectural disruption	Extensive infiltration of the stratum basale, thinning of the mucous membrane, sever edema	Three ulcers	Three lymph nodes
4	Extensive crypt loss, ulceration	Extensive infiltration of the submucosal layer	More than three ulcers	More than three lymph nodes

## Data Availability

Data are available upon request.

## Supplementary Materials

The following supporting information can be downloaded at: https://www.mdpi.com/article/10.3390/jcm13226661/s1, Table S1: Nutritional facts of Oxepa and Ensure Plus; Table S2: Disease Activity Index (DAI) values; Table S3: Microscopic activity index (MAI) values; Table S4: Degree of presence of MPO-positive cells (neutrophils) in the large intestine (per mm^2^); Table S5: Degree of presence of CD68-positive cells (macrophages) in the large intestine (per mm^2^); Table S6: TNF-α (pg/mL) values in serum taken from inferior vena cava and portal vein; Table S7: IL-17 (pg/mL) values in serum taken from inferior vena cava and portal vein; Table S8: TNF-α (pg/g) values in different tissues; Table S9: IL-17 (pg/g) values in different tissues; Table S10: Degree of presence of MPO-positive cells (neutrophils) in the liver, spleen, and lung; Table S11: Degree of presence of CD68-positive cells (macrophages) in the liver, spleen, and lung.
