# Supplementary material for: The Combined Administration of Eicosapentaenoic Acid (EPA) and Gamma-Linolenic Acid (GLA) in Experimentally Induced Colitis: An Experimental Study in Rats
Source: J Clin Med. 2024 Nov 6;13(22):6661. doi: 10.3390/jcm13226661 (PMC11594508; doi:10.3390/jcm13226661)
Supplement: Supplementary file 1 [file jcm-13-06661-s001.zip › jcm-3192153-supplementary.pdf]

Table S1. Nutritional facts of Oxepa and Ensure Plus

Features	Oxepa	Ensure Plus
Calorie yield, kilocalories / milliliter	1.5	1.5
Proteins, Calories%	16.7	15
Fat, Calories%	55.2	28
Carbohydrates, calories%	25:75	57
Total Calories: grams of nitrogen	28.1	168: 1
Non-Protein Calories: Nitrogen	150: 1	146: 1
Osmolality, mOsm / kg H2O	125: 1	680
Osmolality by volume, mOsm / Liter	535	509
Kidney load, mOsm / Liter	384	430

Nutritional facts (per liter)	Oxepa	Ensure Plus
Proteins, grams	62.5	62.5
Fat, grams	93.8	49.2
Carbohydrates, grams	105.3	202
L-Carnitine, milligrams	185	-
Taurine, milligrams	320	-
Water, grams	785	774.3
Energy, Kilometers	1500	1500

Vitamins (per liter)	Oxepa	Ensure Plus
Vitamin A, micrograms palmitate	1580	880
Beta-Carotene, micrograms	670	290
Vitamin D, micrograms	11	20
Vitamin E, milligrams	210	21
Vitamin K, micrograms	100	120
Vitamin C, milligrams	840	120
Folic acid, micrograms	420	400
Thiamine (Vitamin B1), milligrams	3.2	2
Riboflavin (Vitamin B2), milligrams	3.6	2.7
Vitamin B6, milligrams	4.3	2.7
Vitamin B12, micrograms	6	5.5
Niacin, milligrams	30	26
Choline, milligrams	640	550
Biotin, micrograms	60	60
Pantothenic acid, milligrams	13	11

Minerals (per liter)	Oxepa	Ensure Plus
Sodium, milligrams	1310	920
Sodium, milliequivalents	57.0	-
Potassium, milligrams	1960	16000
Potassium, milliequivalents	50.1	-
Chlorine, milligrams	1690	1100
Chlorine, milliequivalents	47.7	-
Calcium, milligrams	1060	1200
Phosphorus, milligrams	1000	1000
Magnesium, milligrams	320	300
Iodine, micrograms	160	220
Manganese, milligrams	5.3	5
Copper, milligrams	2.2	1.8
Zinc, milligrams	18	18
Iron, milligrams	20	21
Selenium, micrograms	77	83
Chromium, micrograms	130	75
Molybdenum, micrograms	160	160

Table S2. Disease Activity Index (DAI) values.

DSS group	Ensure Plus group	Oxepa group	p-value
10.37 ± 1.18	10.28 ± 1.88	9.57 ± 2.14	p=0.538

Table S3. Microscopic Activity Index (MAI) values.

DSS group	Ensure Plus group	Oxepa group	p-value
13.75 ± 1.67	10.28 ± 3.16*	7.29 ± 2.56*,#	<0.001

Values with significant difference from DSS group are marked with an asterisk (*) and values with significant difference from Ensure Plus group are marked with a hash (#).

Table S4. Degree of presence of MPO positive cells (neutrophils) in the large intestine (per mm^2).

	DSS group	Ensure Plus group	Oxepa group	p-value
**Large Intestine**	165.02 ± 37.54	135.6 ± 41.73	89.64 ± 13.68*,#	<0.001

Values with significant difference from Ensure Plus group are marked with an asterisk (*).

Table S5. Degree of presence of CD68 positive cells (macrophages) in the large intestine (per mm^2).

	DSS group	Ensure Plus group	Oxepa group	p-value
**Large Intestine**	12.53 ± 4	17.53 ± 8.84	23.16 ± 9.91*	0.038

Values with significant difference from DSS group are marked with an asterisk (*).

Table S6. TNF-α (pg/ml) values in serum taken from inferior vena cava and portal vein.

	DSS group	Ensure Plus group	Oxepa group	p-value
**IVC**	1835 ± 2649	15034 ± 28027	32933 ± 44787	0.280
**PV**	4352 ± 7681	23586 ± 36380	23308 ± 35577	0.315

Table S7. IL-17 (pg/ml) values in serum taken from inferior vena cava and portal vein.

	DSS group	Ensure Plus group	Oxepa group	p-value
**IVC**	62.5 ± 85.31	103.5 ± 113.85	22.22 ± 22.37	0.134
**PV**	120 ± 126.88	214.37 ± 310.43	29.44 ± 22.14	0.157

Table S8. TNF-α (pg/g) values in different tissues.

	DSS group	Ensure Plus group	Oxepa group	p-value
**Large Intestine**	165.77 ± 45.07	99.18 ± 24.67	86.9 ± 47.47	0.160
**Liver**	100.50 ± 67.89	24.13 ± 47.13	21.15 ± 55.17	0.383
**Spleen**	41.05 ± 50.72	49.44 ± 46.58	72.21 ± 52.43	0.836
**Lung**	12.77 ± 17.51	15.32 ± 21.66	16.61 ± 23.49	0.671

Table S9. IL-17 (pg/g) values in different tissues.

	DSS group	Ensure Plus group	Oxepa group	p-value
**Large Intestine**	121.33 ± 37.07	45 ± 42.63*	54 ± 29.83*	0.050
**Liver**	123.66 ± 66.68	416.4 ± 234.39	379 ± 135.54	0.116
**Spleen**	10.01 ± 2.03	11.33 ± 2.3	41.75 ± 61.51	0.522
**Lung**	116 ± 32.96	38.8 ± 34.33	96.75 ± 55.81	0.066

Values with significant difference from DSS group are marked with an asterisk (*).

Table S10. Degree of presence of MPO positive cells (neutrophils) in the liver, spleen and lung.

	DSS group	Ensure Plus group	Oxepa group	p-value
**Liver**	1 ± 0.76	1.71 ± 0.76	1.14 ± 0.89	0.227
**Spleen**	1.75 ± 0.5	1.63 ± 0.52	1.43 ± 0.53	0.594
**Lung**	1.9 ± 0.32	1.8 ± 0.42	1.22 ± 0.44*,#	<0.001

Values with significant difference from DSS group are marked with an asterisk (*) and values with significant difference from Ensure Plus group are marked with a hash (#).

Table S11. Degree of presence of CD68 positive cells (macrophages) in the liver, spleen and lung.

	DSS group	Ensure Plus group	Oxepa group	p
**Liver**	0.25 ± 0.46	0.28 ± 0.48	0	0.348
**Spleen**	3.2 ± 0.42	3.5 ± 0.7	2.22 ± 0.44$^{*,\#}$	0.002
**Lung**	1	1	1.11 ± 0.33	0.341

Values with significant difference from DSS group are marked with an asterisk (*) and values with significant difference from Ensure Plus group are marked with a hash (#).
